# Customized Multilevel 3D Printing Implant for Reconstructing Spine Tumor: A Retrospective Case Series Study in a Single Center

**DOI:** 10.1111/os.13357

**Published:** 2022-07-27

**Authors:** Zhengwang Sun, Mengchen Yin, Yueli Sun, Mo Cheng, Meng Fang, Wending Huang, Junming Ma, Wangjun Yan

**Affiliations:** ^1^ Department of Musculoskeletal Surgery, Shanghai Cancer Center Fudan University Shanghai China; ^2^ Department of Orthopaedics, Longhua Hospital Shanghai University of Traditional Chinese Medicine Shanghai China

**Keywords:** customized 3D printing model, implant subsidence, multilevel total en bloc spondylectomy, reconstructing, spine tumor

## Abstract

**Objective:**

To investigate the clinical efficacy and safety of 3D printed artificial vertebral body for patients who underwent multilevel total en bloc spondylectomy (TES) and analyze whether it could reduce the incidence of implant subsidence.

**Methods:**

This is a retrospective study. From January 2017 to May 2018, eight consecutive cases with spine tumor undergoing multilevel TES were analyzed. All patients underwent X‐ray and CT examinations to evaluate the stability of internal fixation during the postoperative follow‐up. Demographic, surgical details, clinical data, and perioperative complications was collected. Visual analog scale, Frankel score, and spinal instability neoplastic score (SINS) classification were also recorded.

**Results:**

There were six cases of primary spinal tumor and two cases of metastatic spinal tumor. All patients achieved remarkable pain relief and improvement in neurological function. Five patients underwent operation through the posterior approach, one patient underwent operation through the anterior approach and the remaining two patients through a combined anterior and posterior approach. At the last follow‐up period, X‐rays showed that the 3D printed artificial vertebral body of all cases matched well, and the fixation was reliable. Hardware failure such as loosening, sinking, breaking, and displacement wasn't observed during the follow‐up period.

**Conclusion:**

3D printed customized artificial vertebral body can provide satisfying good clinical and radiological outcomes for patients who have undergone multilevel TES.

## Graphical abstract

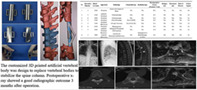

1. The customized 3D printed artificial vertebral body was design to replace vertebral bodies to stabilize the spine column. Postoperative X‐ray showed a good radiographic outcome 3 months after operation.

2. Treatment and outcomes of eight patients who underwent multilevel TES.

3. A presentation of a typical case.

## Introduction

Spinal tumors are mainly divided into primary tumors and metastatic tumors. Primary spinal tumors are rare, accounting for about 4.6% to 8.8% of all bone tumors, and spinal metastases account for 50% of all bone metastases.^1^, ^2^, ^3^ It has been reported in the literature that 10% to 30% of patients with primary malignant tumors will have spinal metastasis at an advanced stage.^4^, ^5^

The main goal of surgical treatment is to remove the tumor, relieve nerve compression and rebuild the stability of the spine. For patients with primary malignant tumors and long‐lived metastatic tumors of the spine, total en bloc spondylectomy (TES) can remove the tumor as a whole, thereby achieving a safe tumor‐free border and reducing recurrence.^6^, ^7^, ^8^ This technology has been proven to be effective in local tumor control and improves the survival rate of patients with spinal tumors. However, how to complete the reconstruction of the spine after tumor resection is still a difficult problem.

In 1986, Harms first used titanium mesh to reconstruct the anterior column structure by bone grafting of the ilium, fibula, and tibia to avoid complications such as fracture and collapse. Since then, it has become a standard technique for vertebral body resection and reconstruction. However, follow‐up of a large number of cases showed that due to the cutting effect of the titanium mesh, its sinking rate is high, which easily leads to reconstruction failure. The incidence of titanium mesh sinking was from 16% to 36%. Furthermore, the failure rate of internal fixation failure of patients after TES was much higher. Therefore, how to provide reliable spinal stability for patients with multilevel TES is challenging and meaningful.^9^, ^10^, ^11^, ^12^

3D printing is an important technology that emerged in the 1980s. It is different from the traditional cutting and casting technology.^13^ It not only changes the physical structure of the product but can also be customized according to individual needs to achieve a complete match between the material and the diseased part. The 3D printed prostheses can complete the completeness and functional reconstruction of defects in complex anatomical structures, and have been used in maxillofacial surgery reconstruction, neurosurgery skull reconstruction, and anterior column reconstruction after spinal tumor resection.^14^, ^15^ The customized 3D printed artificial vertebral body has a better individualized design. Compared with the titanium mesh, it has a larger contact area, making it more closely attached to the upper and lower adjacent vertebral bodies, reducing the probability of settlement due to cutting. The micro‐hole design that simulates the trabecular bone structure has an elastic modulus similar to that of cancellous bone, which is more conducive to the growth of bone tissue and achieves the purpose of fusion.^16^

Since 2017, our center has tried to use 3D printed artificial vertebral body to reconstruct the column after multilevel TES. The purpose of study was to: (i) investigate the clinical efficacy of prosthesis; (ii) analyze whether 3D printing artificial vertebral body can reduce the incidence of implant subsidence; (iii) investigate the safety of 3D printed artificial vertebral body.

## Methods

### Patient Selection

The patients were chosen according to the following inclusion and exclusion criteria. Inclusion criteria included: (i) patients diagnosed with spinal primary malignant tumors or metastatic tumors; (ii) 3D printed artificial vertebral body reconstruction treatment with TES surgery; (iii) expected survival was over 6 months. Exclusion criteria included: (i) patients with malignant metastatic tumors who cannot tolerate surgery or undergo palliative surgery; (ii) patients with primary spinal benign tumors; (iii) having metastases in the lungs or other organs; (iv) survival is expected to be less than 6 months.

In this single‐center, retrospective study, we analyzed eight consecutive patients who underwent multilevel TES using 3D printing implant for reconstruction who were enrolled from January 2017 to May 2018. The work has been in line with the STROCSS criteria. Informed consent was obtained from all the patients. The study protocol was approved by the institutional review board of the hospital (IRB Number: FDRC‐STDI‐2017‐03‐341).

### Design of Customized Multilevel 3D Printing Implant

Before establishing surgical procedures, patients received contrast‐enhanced CT and MRI imaging data to assess the vertebral destructions, and the relationship between the tumor and the surrounding neurovascular structures. Three‐dimensional printed models were constructed based mainly on the CT angiography data set (DICOM format) by using Mimics software 16.0 (Materialize Inc., Leuven, Belgium). The radius of the adjacent vertebral body is reduced by 1–2 mm as the radius of the 3D printed artificial vertebral body, the distance between the upper and lower adjacent vertebral bodies is used as the height of the artificial vertebral body. The curvature of prosthesis is designed according to the position of the tumor and the physiological curvature. Then, the artificial vertebral body is printed after grid and porous processing.

The vertebral body adopts a cylindrical design, the diameter of the thoracic vertebral artificial vertebral body is 15 × 21 mm, and the diameter of the lumbar vertebral artificial vertebral body is 18 × 24 mm. The height of 3D printed artificial vertebrae is designed according to the height of the diseased vertebrae measured by CT. The angle between the upper and lower contact surfaces and the horizontal is divided into three models: 0°, 4°, and 8°. The 3D printed artificial vertebral body adopts a simulated vertebral cancellous bone trabecular structure, with a pore diameter of 600–800 μm and a porosity of 70%–80%. The prosthesis is made of Ti6Al4V, the device used is EOSm280 (Naton Technology Group, Beijing, China). Three duplications of slightly different sizes were prepared in advance to guarantee optimal placement in the operation.

### Evaluation Methods and Indicators

#### Clinical Outcome

The patients were followed up on the outpatient basis or *via* telephone interviews every month for the first 3 months, and every 3 months for the next 2 years. The length of the follow‐up period was defined as the date of surgery to the date of tumor‐related death or May 2021. Perioperative complications were defined as those occurring from the first day after operation to the date of discharge.

#### Visual Analog Scale

Visual analog scale (VAS) was used to evaluate pain control. Neurological status was assessed using the Frankel score, before surgery and at final follow up. Spinal instability neoplastic score (SINS) classification was used to diagnose neoplastic spinal instability. All details including potential benefits as well as risks and complications about the surgical procedure were also assessed.

VAS is a reliable and valid measurement of pain. It has a horizontal, 100 mm‐long line, with “no pain” recorded on the left side (score: 0) and “pain as bad as it could be” on the right side (score: 10).

#### SINS Classification

SINS classification comprises six individual component scores: spine location, pain, lesion bone quality, radiographic alignment, vertebral body collapse, and posterolateral involvement of the spinal elements. The total score is divided into three categories in terms of stability: stable (0–6 points), potentially unstable (7–12 points), and unstable (13–18 points). A surgical consultation is recommended for patients with SINS scores greater than 7.

#### Frankel Grade

The Frankel grade classification provides an assessment of spinal cord function and is used as a tool in spinal cord injury. The grades are as follows: Grade A—Complete neurological injury: No motor or sensory function detected below level of lesion. Grade B—Preserved sensation only: No motor function detected below level of lesion; some sensory function below level of lesion preserved. Grade C—Preserved motor, nonfunctional: Some voluntary motor function preserved below level of lesion but too weak to serve any useful purpose; sensation may or may not be preserved. Grade D—Preserved motor, functional: Functionally useful voluntary motor function below level of injury is preserved. Grade E—Normal motor function: Normal motor and sensory function below level of lesion; abnormal reflexes may persist.

### Radiological Data

All patients underwent X‐ray and CT examinations to evaluate the stability of internal fixation and fusion rate during the postoperative follow‐up. The 3D printed artificial vertebral body dropping more than 3 mm is regarded as sinking.

### Statistical Analysis

All statistical analyses were performed with the statistical program SPSS version 22.0 (SPSS Inc., Chicago, IL, USA). Continuous variables were expressed as mean ± SD.

## Results

### General Results

There were six cases of primary spinal tumor and two cases of metastatic spinal tumor. Our series comprised four males and four females, with a mean age of 34.5 (range 22–51) years. The duration of symptoms averaged 7.6 months (range 1 to 36 months). The most frequent location was the thoracic spine (n = 6), followed by the cervicothoracic spine (n = 2). Two patients were admitted into our institution for secondary operations, and the interval between initial surgery and recurrence were 12 and 20 months, respectively. The most common complaint at presentation was back pain (n = 8, 100%, VAS score range 5–8), followed by extremity numbness and weakness (n = 7, 87.5%, Frankel B to D). In addition, three patients experienced incontinence (Table 1).

**TABLE 1:** The general information of eight patients underwent multilevel total en bloc spondylectomy

No	Gender	Age	Tumor statue	Tumor location	Main symptoms	Duration of symptom (m)	Treatment history	Preoperative scores (Frankel/ VAS)	Sphincter disturbance	WBB staging	SINS scores
1	M	53	Primary	T_9_‐T_11_	Paralysis of lower limbs and back pain	1	None	C/7	Yes	3–11, A‐D	13
2	M	51	Primary	C_6_‐T_2_	Neck pain	36	None	C/4	No	2–11, A‐D	8
3	M	44	Primary	T_8_‐T_12_	Back pain, paralysis of lower limb	1	Biopsy, chemotherapy	B/6	Yes	1–7, A‐D	9
4	F	28	Metastasis	T_4_‐T_6_	Numbness and back pain	3	Thoracoscopic resection of the tumor	D/5	No	2–11, A‐D	10
5	F	37	Primary	T_2_‐T_4_	Paralysis of lower limbs and back pain	5	Partial tumor resection, chemotherapy, radiotherapy	D/8	No	1–12, A‐D	11
6	M	28	Primary	T_6_‐T_8_	Back pain, Paralysis of lower limbs	4	None	B/6	Yes	2–11, A‐D	13
7	F	22	Metastasis	T_4_‐T_7_	Back pain, paralysis of lower limb	3	Partial resection of mediastinal tumor	D/5	No	1–12, A‐D	10
8	F	31	Primary	C_5_‐T_1_	Right upper limb paralysis and pain	8	C6 Vertebral Body subtotal resection	D/8	No	1–11, A‐D	14

### Intraoperative Results

**Fig. 1:**
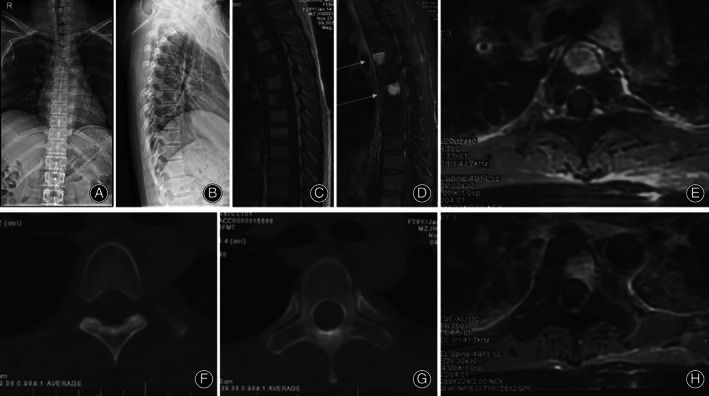
(A, B) Preoperative frontal and lateral radiograph X‐ray. (C–H) Preoperative MRI T1, T2 weighted images showing abnormal signal in thoracic 4–6 vertebral body. Preoperative CT showing slight reduction in vertebral density.

**Fig. 2:**
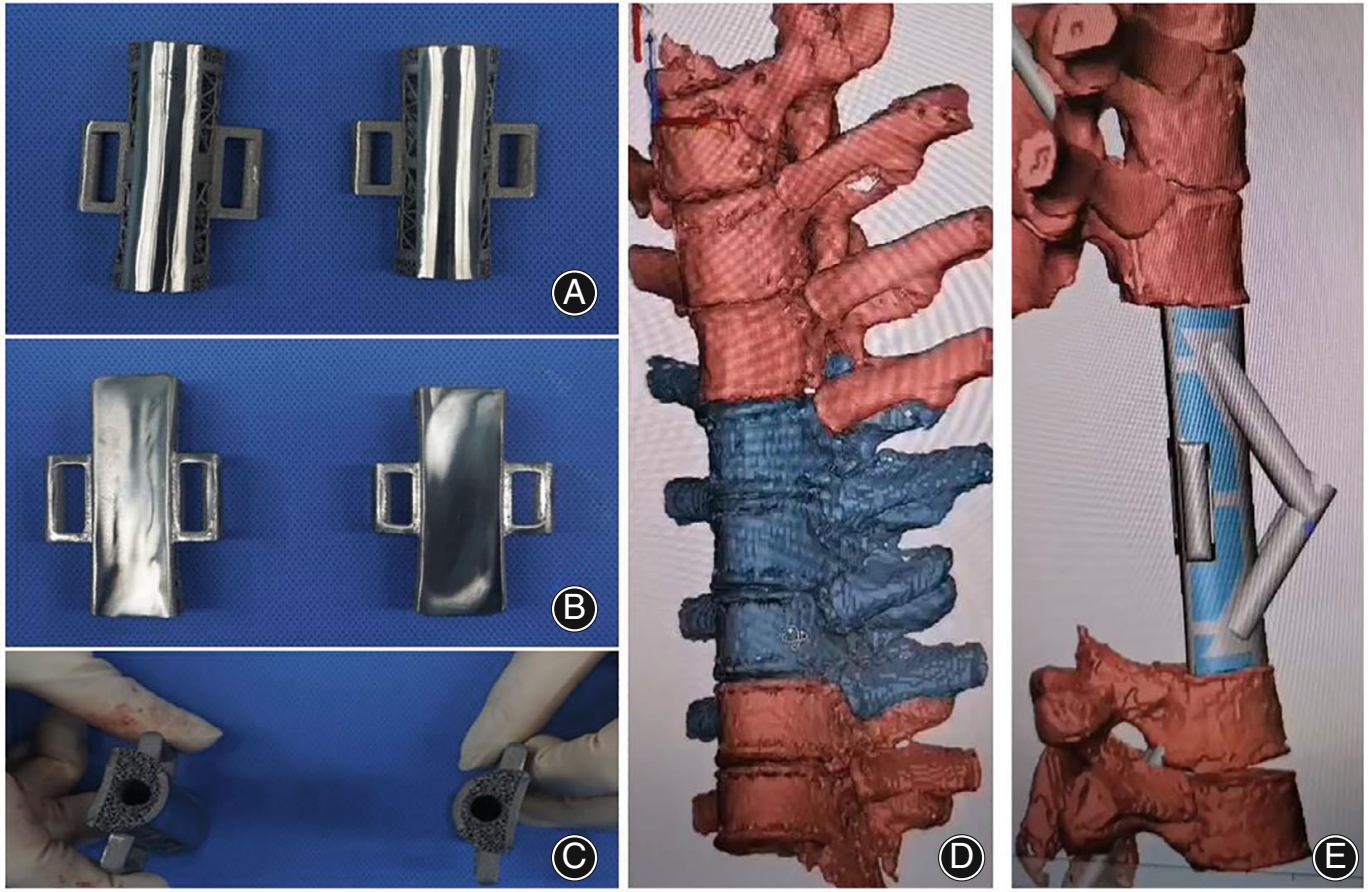
(A–C) A physical view of the 3D prosthesis. (D, E) Preoperative 3D printed prosthesis designs to replace vertebral bodies to stabilize the spine column.

**Fig. 3:**
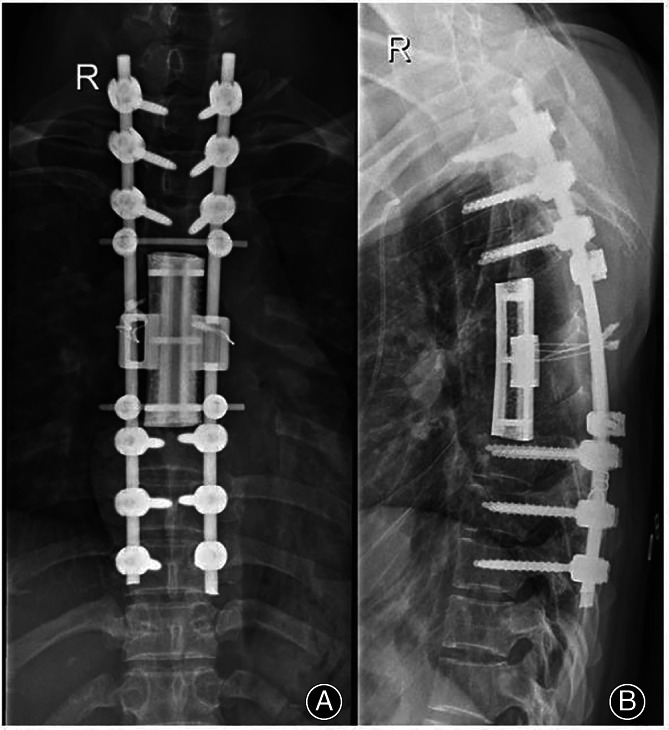
(A, B) Postoperative X‐ray review results about 3 months after operation.

Five patients underwent operation through the posterior approach, one patient underwent operation through the anterior approach and the remaining two patients through a combined anterior and posterior approach. Radical resection was performed in all the eight patients. Representative images are provided in Figures 1, 2, 3. The surgical time averaged 4.8 (range 3–8) hours with a mean blood loss of 2700 (range 1500–6500) mL. Postoperative adjuvant therapies included concurrent chemotherapy therapy and radiotherapy in two patients, chemotherapy alone in three patients and intraoperative radiotherapy (12GY) in one patient. The drugs for chemotherapy were mainly COVP and VAC/IE. Pathologically, the six patients with primary lesions were diagnosed as having the giant cell tumor of bone (n = 1), osteosarcoma (n = 2), Ewing's sarcoma (n = 1), hemangioma (n = 1), and epithelioid hemangioendothelioma (n = 1), and the other two patients with metastatic lesions were diagnosed as having mediastina.

### Clinical Outcome

The mean preoperative VAS score was 6.7. The preoperative Frankel grade was C or even worse. All patients achieved remarkable pain relief (mean postoperative VAS score range 1.8) and improvement in neurological function (postoperative Frankel grade D or E). Two patients developed metastasis at an average of 8 months after surgery. Two patients died, with mean survival duration of 21 months (Table 2).

**TABLE 2:** Treatment and outcomes of eight patients who underwent multilevel total en bloc spondylectomy

No	Operative time (h)	Blood loss (mL)	Approach	Pathology	Chemotherapy	Radiotherapy	Postoperative scores (Frankel/VAS)	Relapse (m)	Metastasis (m)	Died of disease	Follow up (m)
1	3	2000	Posterior	Giant cell tumor of bone	No	No	D/1	No	No	No	40
2	5	1500	Anterior–Posterior	Osteosarcoma	Yes	No	E/1	No	No	No	24
3	8	3000	Posterior	Osteosarcoma	Yes	No	E/2	No	Yes	Yes	24
4	5	2300	Posterior	Neuroblastoma	Yes (COVP)	Yes	E/2	No	No	No	20
5	5	2300	Posterior (Revision)	Ewing's Sarcoma	Yes (VAC/IE)	Yes	E/1	No	Yes	Yes	18
6	6	6500	Posterior	Hemangioma	No	No	E/1	No	No	No	24
7	5	2000	Anterior–Posterior	Malignant peripheral Schwannoma	Yes	No	E/0	No	No	No	24
8	5	2000	Posterior‐ Anterior (revision)	Epithelioid hemangioendothelioma	No	Intraoperative radiotherapy (12GY)	E/1	No	No	No	24

### Radiological Outcome

At the last follow‐up period, X‐rays showed that the 3D printed artificial vertebral body of all cases matched well with the adjacent upper and lower vertebral bodies, and the fixation was reliable. Hardware failure such as loosening, sinking, breaking, and displacement wasn't observed during the follow‐up period.

### Complications

Cerebrospinal fluid leakage occurred in two cases. After repair, they were sealed with gelatin sponge, and drainage was continued after the operation. The drainage tube extubation time was prolonged to 1 week after operation. They were cured after symptomatic treatment such as local compression. Two patients with intercostal neuralgia received no special treatment, and the pain relieved 1 month after the operation.

## Discussion

### Application of 3D Printed Prosthesis for Multilevel TES

For reconstruction of bone defects after multilevel TES, 3D printing artificial vertebral bodies could greatly reduce the risk of prosthesis collapse and loosening, and its advantages were more significant. The customized requirements made it possible to reconstruct complex and multilevel spinal bone defects with individualized artificial vertebral bodies.^13^, ^14^, ^15^, ^16^ Yoshioka reported on the clinical outcome of 22 patients for reconstructions after three or more levels of TES.^11^ Cage subsidence (>2 cm) was a common phenomenon (50%) and already observed 1 month after surgery in eight of the 11 cases. Girolami evaluated 13 patients using custom‐made 3D‐printed titanium prosthesis after en bloc resection for spinal tumor. At an average 10‐month follow‐up, only one patient with severity of the subsidence led to revision of the construct. Only one implant was removed due to local recurrence of the disease and one was revised due to progressive distal junctional kyphosis. The study indicated that 3D printing could be effectively used to produce custom‐made prosthesis for anterior column reconstruction.^17^ Choy designed a 3D printed prosthesis that can accommodate two pedicle screws and used it for spine reconstruction.^18^

### 3D Printed Prosthesis Can Provide Satisfactory Stability

For reconstruction of irregular vertebral body defects in special locations, 3D printing artificial vertebral bodies also had its unique advantages. Mobbs reported a case of C2 chordoma after resection, and reconstruction with individualized artificial vertebral body. There was no loosening and displacement of the prosthesis during the 9‐month follow‐up after the operation, achieving osseointegration. Xu reported a case of C2 Ewing's sarcoma after resection, which was reconstructed with a personalized artificial vertebral body. There was no local recurrence 1 year after operation, no distant metastasis, and osseointegration of the artificial vertebral body and adjacent vertebral bodies.^19^ Kim reported a patient with sacral osteosarcoma who achieved accurate reconstruction of the semi‐sacrum through a 3D printed prosthesis. The prosthesis was in good position 1 year after surgery and osseointegrated with the surrounding bone.^20^ Guo reported a case of sacral chordoma using a personalized artificial sacrum to reconstruct the sacral defect. Eight months after the operation, the prosthesis was broken, but the patient had no discomfort, and the activity was still good for 1 year after the operation, and there was no feeling of sacroiliac joint instability.^21^

The 3D printed prosthesis in this study was designed to simulate human cancellous bone trabecular structure with a porosity of 80%. Parthasarathy believed that the strength of a porous implant depended on its volume and porosity. When bone grew into the implant, it became a “reinforced concrete structure,” and its compressive strength increased significantly.^22^ The elastic modulus of the implant was more important and played a key role in the early bone fusion process.

The porous structure of the 3D printed artificial vertebral body was conducive to bone ingrowth. Whether this kind of porous prosthesis had good bone ingrowth ability depends mainly on porosity, pore size, shape, and distribution of pores. Among them, porosity and pore size played a key role in bone ingrowth. Therefore, proper porosity and pore size were the key to whether a porous prosthesis can achieve good bone ingrowth. The 3D printed artificial vertebral body used in this study had a porosity of 80% and a pore diameter of (800 ± 200) μm, which was conducive to the migration and proliferation of bone cells. In addition, the prosthesis adopted an open porous structure with interconnected pores.

### Limitations

This study has some limitations: Firstly, this was a retrospective study, it only studied the application effects of 3D printed individualized artificial vertebrae on spinal tumor resection and reconstruction in a single center. We will further carry out multi‐center research and comparative research on multiple reconstruction methods of the spine. Secondly, the follow‐up time was short, and further evaluation of the long‐term effect was needed. Thirdly, the number of study cases was small, the data obtained was limited, the results may be biased, and it was necessary to obtain more objective clinical results.

### Conclusion

In summary, the new 3D printed customized artificial vertebral body used for spinal reconstruction after total vertebral tumor resection has certain advantages over traditional reconstruction methods. However, no matter which method of reconstruction is based on total spine resection, the indications for surgery are clear, and a reasonable choice of surgical plan is a prerequisite for successful surgery. Through precise preoperative design and fine intraoperative operations, the 3D printed individual artificial vertebral body can finally achieve the precise reconstruction of the spine during the operation, maximize the immediate stability of the spine after reconstruction, and lay a foundation for the final realization of spinal intervertebral fusion basis. 3D printed artificial vertebrae have shown great advantages in spinal reconstruction for multilevel TES, but the current clinical applications are still being explored.

## Funding

The study was supported by Shanghai Municipal Health Commission (202140393).

## Competing Interests

All authors read and approved the final manuscript and declare that they have no competing interests.

## Ethics Approval

The case was reviewed by our hospital's ethics committee and ethical approval was waived.

## Consent to Participate

Written consent was obtained from all participants.

## Consent for Publication

Written patient consent was obtained for publication of all aspects of the case including personal and clinical details and images, which may compromise anonymity.

## Author Contribution

SZW, YMC, and SYL are co‐first authors of this manuscript, contributing equally to the design, conduct of, and drafting the manuscript. All authors participated in the design and performed the study. MJM and YWJ are corresponding authors of this manuscript.

## Data Availability

All supporting data can be provided upon request to the authors.

## References

[1] Dea N , Gokaslan Z , Choi D , Fisher C . Spine oncology ‐ primary spine tumors. Neurosurgery. 2017;80:S124–30. 28350941 10.1093/neuros/nyw064

[2] Boussios S , Cooke D , Hayward C , et al. Metastatic spinal cord compression: unraveling the diagnostic and therapeutic challenges. Anticancer Res. 2018;38:4987–97. 30194142 10.21873/anticanres.12817

[3] Choi D , Bilsky M , Fehlings M , Fisher C , Gokaslan Z . Spine oncology‐metastatic spine tumors. Neurosurgery. 2017;80:S131–7. 28350950 10.1093/neuros/nyw084

[4] Sciubba DM , Goodwin CR , Yurter A , et al. A systematic review of clinical outcomes and prognostic factors for patients undergoing surgery for spinal metastases secondary to breast cancer. Global Spine J. 2016;6:482–96. 27433433 10.1055/s-0035-1564807 PMC4947406

[5] Richardson ED , Price DK , Figg WD . Significant addition to treatment options for bone metastasis in prostate cancer. Cancer Biol Ther. 2012;13:69–70. 22336908 10.4161/cbt.13.2.18441 PMC3342941

[6] Kawahara N , Tomita K , Murakami H , Demura S . Total en bloc spondylectomy for spinal tumors: surgical techniques and related basic background. Orthop Clin North Am. 2009;40(47–63):vi. 10.1016/j.ocl.2008.09.004 19064055

[7] Amendola L , Cappuccio M , De Iure F , Bandiera S , Gasbarrini A , Boriani S . En bloc resections for primary spinal tumors in 20 years of experience: effectiveness and safety. Spine J. 2014;14:2608–17. 24561037 10.1016/j.spinee.2014.02.030

[8] Kato S , Murakami H , Demura S , et al. More than 10‐year follow‐up after total en bloc spondylectomy for spinal tumors. Ann Surg Oncol. 2014;21:1330–6. 24150193 10.1245/s10434-013-3333-7

[9] Chen Y , Chen D , Guo Y , et al. Subsidence of titanium mesh cage: a study based on 300 cases. J Spinal Disord Tech. 2008;21:489–92. 18836360 10.1097/BSD.0b013e318158de22

[10] Grob D , Daehn S , Mannion AF . Titanium mesh cages (TMC) in spine surgery. Eur Spine J. 2005;14:211–21. 15340827 10.1007/s00586-004-0748-7 PMC3476740

[11] Yoshioka K , Murakami H , Demura S , et al. Clinical outcome of spinal reconstruction after total en bloc spondylectomy at 3 or more levels. Spine (Phila pa 1976). 2013;38:E1511–6. 23921327 10.1097/BRS.0b013e3182a6427a

[12] Lau D , Song Y , Guan Z , La Marca F , Park P . Radiological outcomes of static vs expandable titanium cages after corpectomy: a retrospective cohort analysis of subsidence. Neurosurgery. 2013;72:528–39. 10.1227/NEU.0b013e318282a558 23246824

[13] Xiao JR , Huang WD , Yang XH , et al. En bloc resection of primary malignant bone tumor in the cervical spine based on 3‐dimensional printing technology. Orthop Surg. 2016;8:171–8. 27384725 10.1111/os.12234 PMC6584397

[14] Rengier F , Mehndiratta A , von Tengg‐Kobligk H , et al. 3D printing based on imaging data: review of medical applications. Int J Comput Assist Radiol Surg. 2010;5:335–41. 20467825 10.1007/s11548-010-0476-x

[15] Rankin TM , Giovinco NA , Cucher DJ , Watts G , Hurwitz B , Armstrong DG . Three‐dimensional printing surgical instruments: are we there yet? J Surg Res. 2014;189:193–7. 24721602 10.1016/j.jss.2014.02.020 PMC4460996

[16] Waran V , Narayanan V , Karuppiah R , Owen SL , Aziz T . Utility of multimaterial 3D printers in creating models with pathological entities to enhance the training experience of neurosurgeons. J Neurosurg. 2014;120:489–92. 24321044 10.3171/2013.11.JNS131066

[17] Schubert C , van Langeveld MC , Donoso LA . Innovations in 3D printing: a 3D overview from optics to organs. Br J Ophthalmol. 2014;98:159–61. 24288392 10.1136/bjophthalmol-2013-304446

[18] Skelley NW , Smith MJ , Ma R , Cook JL . Three‐dimensional printing technology in orthopaedics. J Am Acad Orthop Surg. 2019;27:918–25. 31268868 10.5435/JAAOS-D-18-00746

[19] Palmquist A , Snis A , Emanuelsson L , Browne M , Thomsen P . Long‐term biocompatibility and osseointegration of electron beam melted, free‐form‐fabricated solid and porous titanium alloy: experimental studies in sheep. J Biomater Appl. 2013;27:1003–16. 22207608 10.1177/0885328211431857

[20] Girolami M , Boriani S , Bandiera S , et al. Biomimetic 3D‐printed custom‐made prosthesis for anterior column reconstruction in the thoracolumbar spine: a tailored option following en bloc resection for spinal tumors: preliminary results on a case‐series of 13 patients. Eur Spine J. 2018;27:3073–83. 30039254 10.1007/s00586-018-5708-8

[21] Choy WJ , Mobbs RJ , Wilcox B , Phan S , Phan K , Sutterlin CE . Reconstruction of thoracic spine using a personalized 3D‐printed vertebral body in adolescent with T9 primary bone tumor. World Neurosurg. 2017;105:1032.e1013–7. 10.1016/j.wneu.2017.05.133 28578109

[22] Xu N , Wei F , Liu X , et al. Reconstruction of the upper cervical spine using a personalized 3D‐printed vertebral body in an adolescent with Ewing sarcoma. Spine (Phila Pa 1976). 2016;41:50–4. 10.1097/BRS.0000000000001179 26335676

